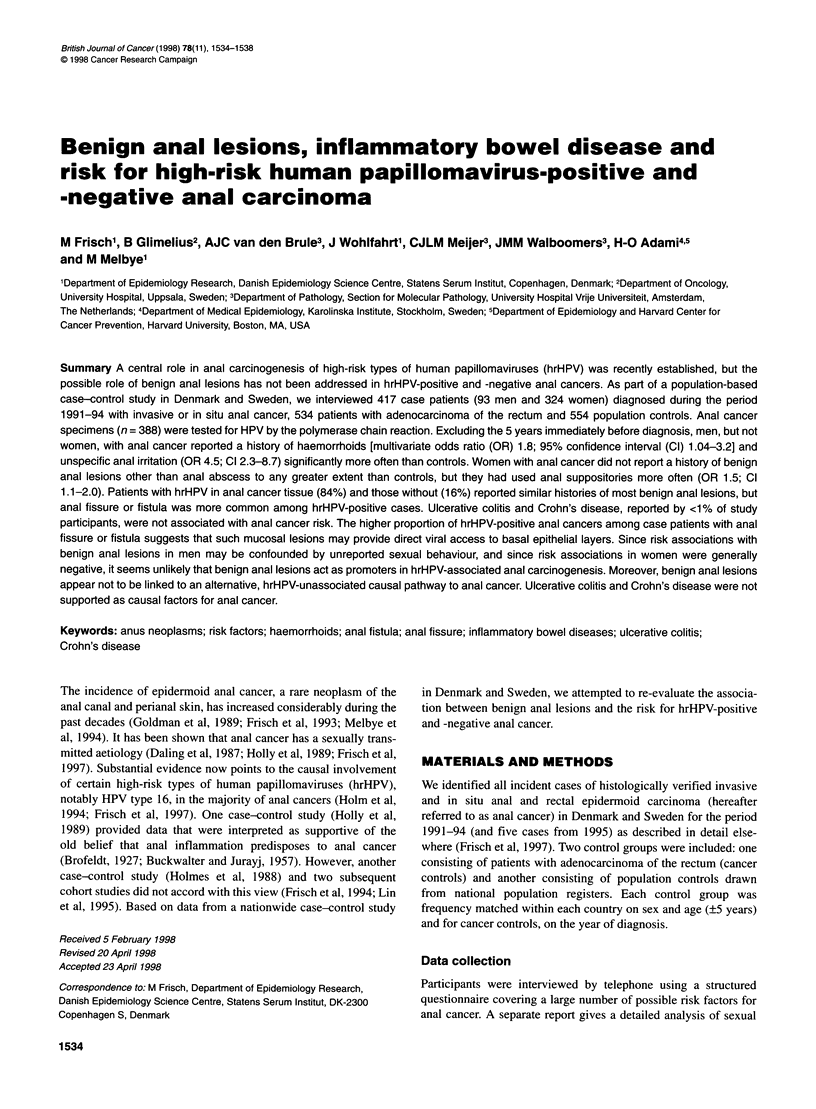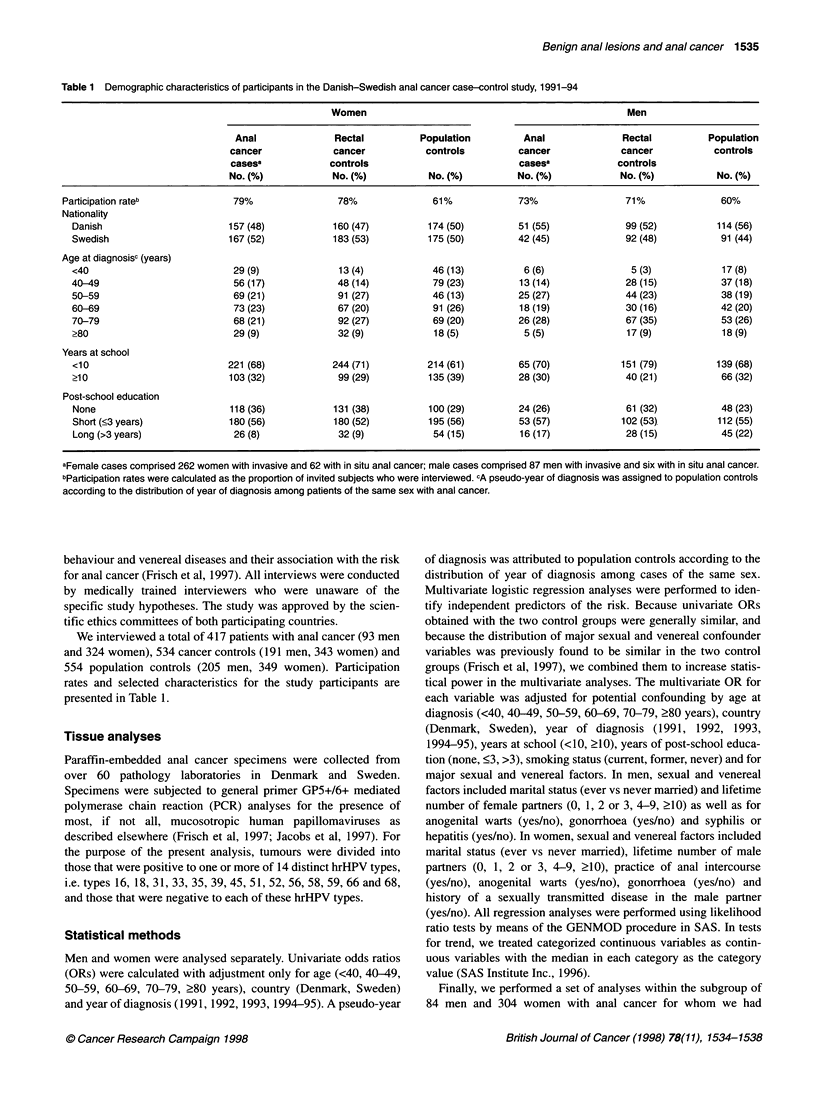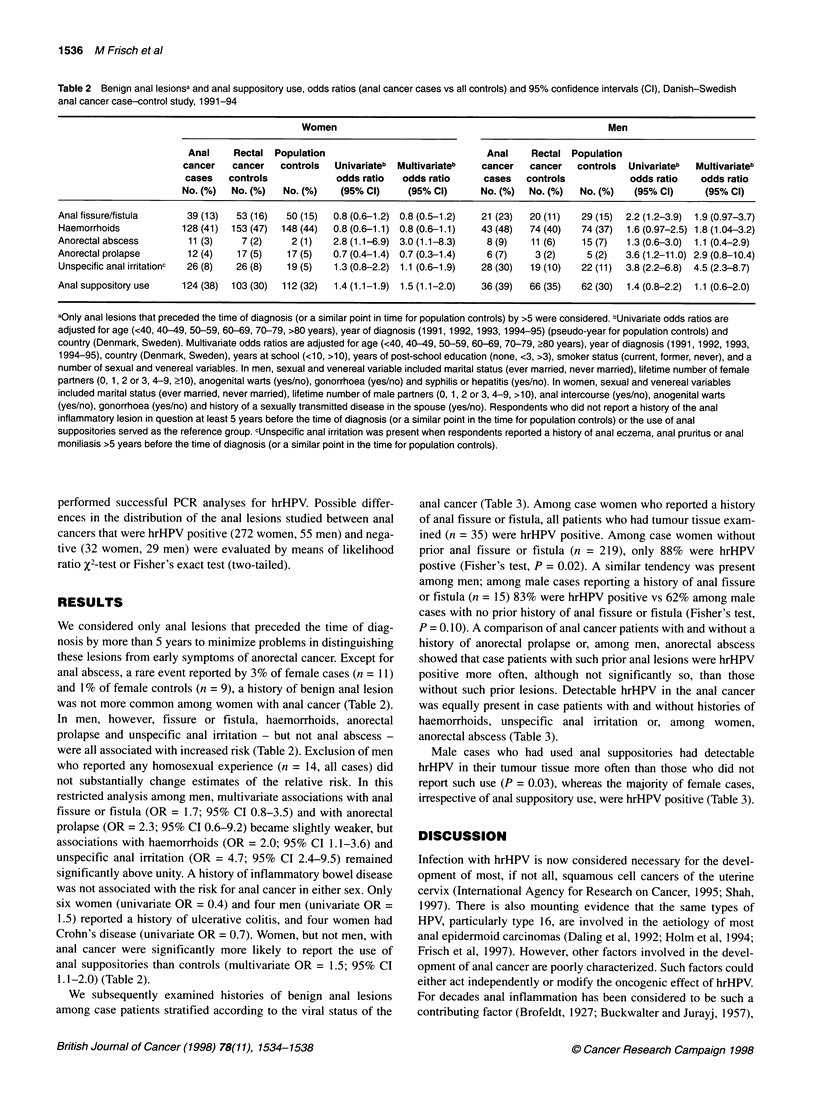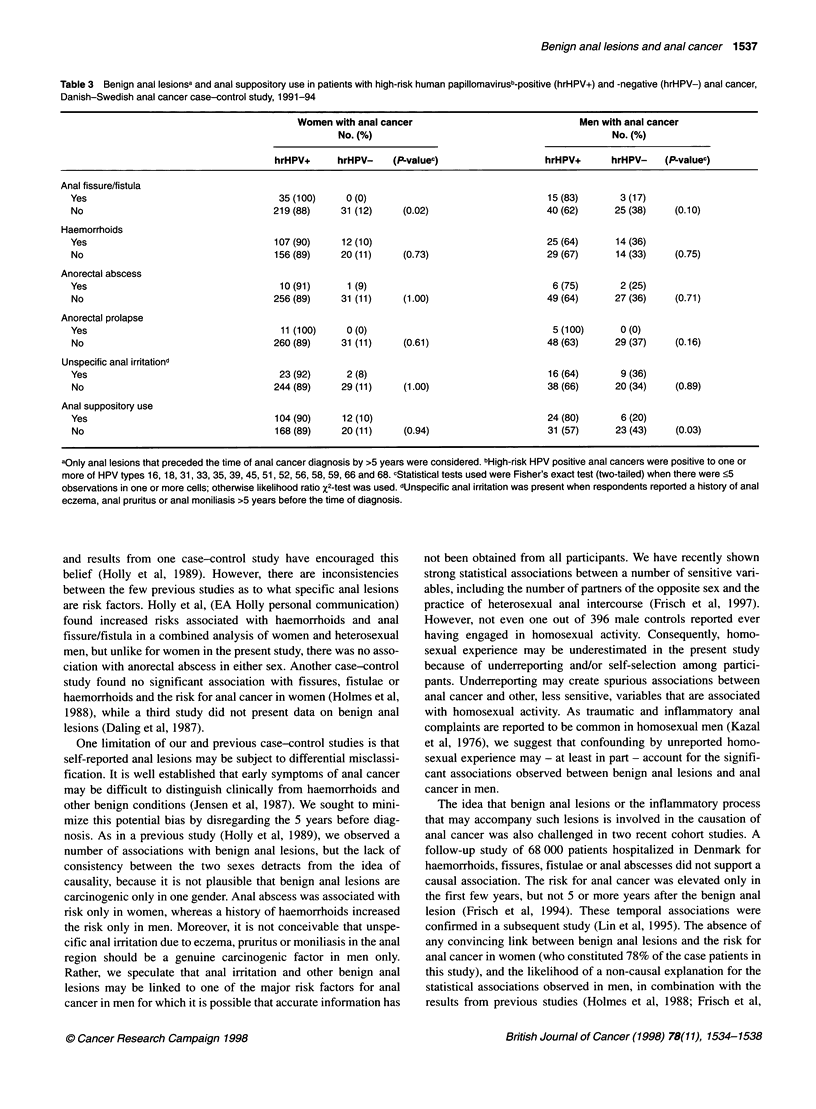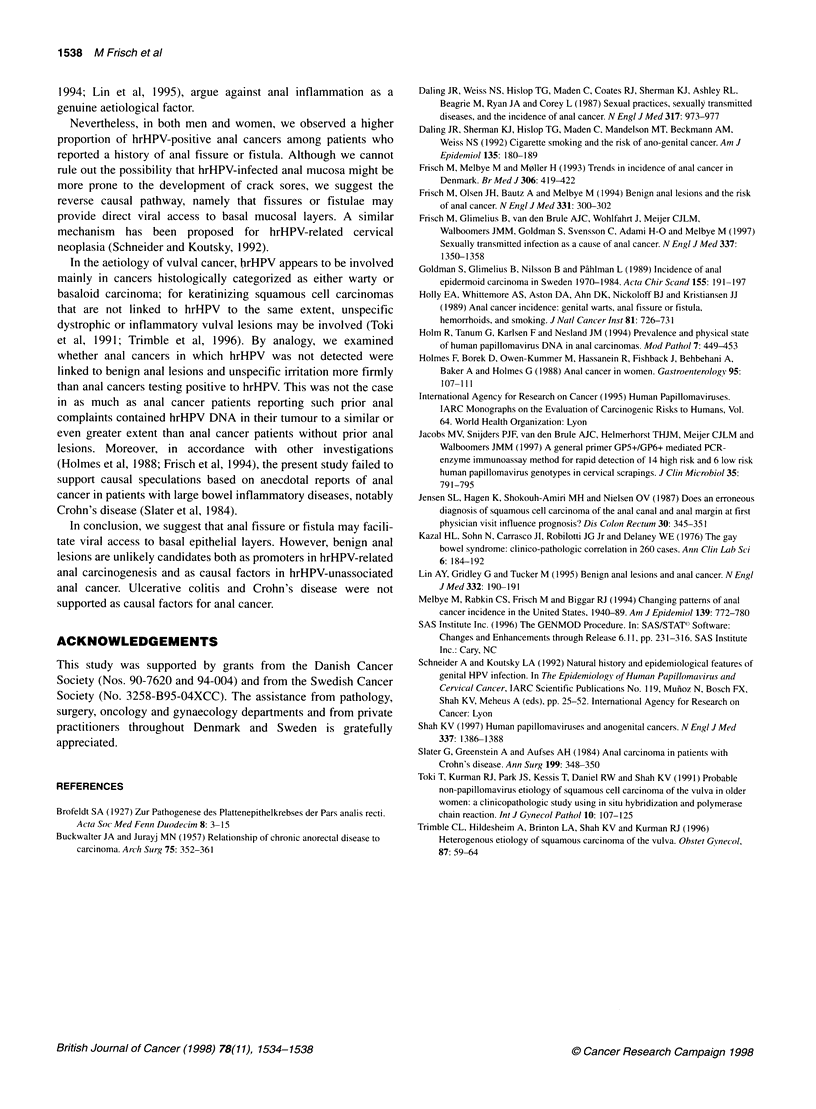# Benign anal lesions, inflammatory bowel disease and risk for high-risk human papillomavirus-positive and -negative anal carcinoma.

**DOI:** 10.1038/bjc.1998.719

**Published:** 1998-12

**Authors:** M. Frisch, B. Glimelius, A. J. van den Brule, J. Wohlfahrt, C. J. Meijer, J. M. Walboomers, H. O. Adami, M. Melbye

**Affiliations:** Department of Epidemiology Research, Danish Epidemiology Science Centre, Statens Serum Institut, Copenhagen.

## Abstract

A central role in anal carcinogenesis of high-risk types of human papillomaviruses (hrHPV) was recently established, but the possible role of benign anal lesions has not been addressed in hrHPV-positive and -negative anal cancers. As part of a population-based case-control study in Denmark and Sweden, we interviewed 417 case patients (93 men and 324 women) diagnosed during the period 1991-94 with invasive or in situ anal cancer, 534 patients with adenocarcinoma of the rectum and 554 population controls. Anal cancer specimens (n = 388) were tested for HPV by the polymerase chain reaction. Excluding the 5 years immediately before diagnosis, men, but not women, with anal cancer reported a history of haemorrhoids [multivariate odds ratio (OR) 1.8; 95% confidence interval (CI) 1.04-3.2] and unspecific anal irritation (OR 4.5; CI 2.3-8.7) significantly more often than controls. Women with anal cancer did not report a history of benign anal lesions other than anal abscess to any greater extent than controls, but they had used anal suppositories more often (OR 1.5; CI 1.1-2.0). Patients with hrHPV in anal cancer tissue (84%) and those without (16%) reported similar histories of most benign anal lesions, but anal fissure or fistula was more common among hrHPV-positive cases. Ulcerative colitis and Crohn's disease, reported by <1% of study participants, were not associated with anal cancer risk. The higher proportion of hrHPV-positive anal cancers among case patients with anal fissure or fistula suggests that such mucosal lesions may provide direct viral access to basal epithelial layers. Since risk associations with benign anal lesions in men may be confounded by unreported sexual behaviour, and since risk associations in women were generally negative, it seems unlikely that benign anal lesions act as promoters in hrHPV-associated anal carcinogenesis. Moreover, benign anal lesions appear not to be linked to an alternative, hrHPV-unassociated causal pathway to anal cancer. Ulcerative colitis and Crohn's disease were not supported as causal factors for anal cancer.


British Journal of Cancer (1998) 78(11), 1534–1538
© 1998 Cancer Research Campaign

### Benign anal lesions, inflammatory bowel disease and risk for high-risk human papillomavirus-positive and -negative anal carcinoma

**M Frisch[1], B Glimelius[2], AJC van den Brule[3], J Wohlfahrt[1], CJLM Meijer[3], JMM Walboomers[3], H-O Adami[4,5] and M Melbye[1]**

[1]Department of Epidemiology Research, Danish Epidemiology Science Centre, Statens Serum Institut, Copenhagen, Denmark; [2]Department of Oncology, University Hospital, Uppsala, Sweden; [3]Department of Pathology, Section for Molecular Pathology, University Hospital Vrije Universiteit, Amsterdam, The Netherlands; [4]Department of Medical Epidemiology, Karolinska Institute, Stockholm, Sweden; [5]Department of Epidemiology and Harvard Center for Cancer Prevention, Harvard University, Boston, MA, USA

**Summary** A central role in anal carcinogenesis of high-risk types of human papillomaviruses (hrHPV) was recently established, but the possible role of benign anal lesions has not been addressed in hrHPV-positive and -negative anal cancers. As part of a population-based case–control study in Denmark and Sweden, we interviewed 417 case patients (93 men and 324 women) diagnosed during the period 1991–94 with invasive or in situ anal cancer, 534 patients with adenocarcinoma of the rectum and 554 population controls. Anal cancer specimens ($n = 388$) were tested for HPV by the polymerase chain reaction. Excluding the 5 years immediately before diagnosis, men, but not women, with anal cancer reported a history of haemorrhoids [multivariate odds ratio (OR) 1.8; 95% confidence interval (CI) 1.04–3.2] and unspecific anal irritation (OR 4.5; CI 2.3–8.7) significantly more often than controls. Women with anal cancer did not report a history of benign anal lesions other than anal abscess to any greater extent than controls, but they had used anal suppositories more often (OR 1.5; CI 1.1–2.0). Patients with hrHPV in anal cancer tissue (84%) and those without (16%) reported similar histories of most benign anal lesions, but anal fissure or fistula was more common among hrHPV-positive cases. Ulcerative colitis and Crohn's disease, reported by <1% of study participants, were not associated with anal cancer risk. The higher proportion of hrHPV-positive anal cancers among case patients with anal fissure or fistula suggests that such mucosal lesions may provide direct viral access to basal epithelial layers. Since risk associations with benign anal lesions in men may be confounded by unreported sexual behaviour, and since risk associations in women were generally negative, it seems unlikely that benign anal lesions act as promoters in hrHPV-associated anal carcinogenesis. Moreover, benign anal lesions appear not to be linked to an alternative, hrHPV-unassociated causal pathway to anal cancer. Ulcerative colitis and Crohn's disease were not supported as causal factors for anal cancer.

**Keywords:** anus neoplasms; risk factors; haemorrhoids; anal fistula; anal fissure; inflammatory bowel diseases; ulcerative colitis; Crohn's disease

The incidence of epidermoid anal cancer, a rare neoplasm of the anal canal and perianal skin, has increased considerably during the past decades (Goldman et al, 1989; Frisch et al, 1993; Melbye et al, 1994). It has been shown that anal cancer has a sexually transmitted aetiology (Daling et al, 1987; Holly et al, 1989; Frisch et al, 1997). Substantial evidence now points to the causal involvement of certain high-risk types of human papillomaviruses (hrHPV), notably HPV type 16, in the majority of anal cancers (Holm et al, 1994; Frisch et al, 1997). One case–control study (Holly et al, 1989) provided data that were interpreted as supportive of the old belief that anal inflammation predisposes to anal cancer (Brofeldt, 1927; Buckwalter and Jurayj, 1957). However, another case–control study (Holmes et al, 1988) and two subsequent cohort studies did not accord with this view (Frisch et al, 1994; Lin et al, 1995). Based on data from a nationwide case–control study in Denmark and Sweden, we attempted to re-evaluate the association between benign anal lesions and the risk for hrHPV-positive and -negative anal cancer.

*Received 5 February 1998*
*Revised 20 April 1998*
*Accepted 23 April 1998*

*Correspondence to:* M Frisch, Department of Epidemiology Research, Danish Epidemiology Science Centre, Statens Serum Institut, DK-2300 Copenhagen S, Denmark

### MATERIALS AND METHODS

We identified all incident cases of histologically verified invasive and in situ anal and rectal epidermoid carcinoma (hereafter referred to as anal cancer) in Denmark and Sweden for the period 1991–94 (and five cases from 1995) as described in detail elsewhere (Frisch et al, 1997). Two control groups were included: one consisting of patients with adenocarcinoma of the rectum (cancer controls) and another consisting of population controls drawn from national population registers. Each control group was frequency matched within each country on sex and age (±5 years) and for cancer controls, on the year of diagnosis.

### Data collection

Participants were interviewed by telephone using a structured questionnaire covering a large number of possible risk factors for anal cancer. A separate report gives a detailed analysis of sexual

**Table 1** Demographic characteristics of participants in the Danish–Swedish anal cancer case–control study, 1991–94

| | Women | | | Men | | |
|---|---|---|---|---|---|---|
| | Anal cancer cases[a] No. (%) | Rectal cancer controls No. (%) | Population controls No. (%) | Anal cancer cases[a] No. (%) | Rectal cancer controls No. (%) | Population controls No. (%) |
| Participation rate[b] | 79% | 78% | 61% | 73% | 71% | 60% |
| Nationality | | | | | | |
| Danish | 157 (48) | 160 (47) | 174 (50) | 51 (55) | 99 (52) | 114 (56) |
| Swedish | 167 (52) | 183 (53) | 175 (50) | 42 (45) | 92 (48) | 91 (44) |
| Age at diagnosis[c] (years) | | | | | | |
| <40 | 29 (9) | 13 (4) | 46 (13) | 6 (6) | 5 (3) | 17 (8) |
| 40–49 | 56 (17) | 48 (14) | 79 (23) | 13 (14) | 28 (15) | 37 (18) |
| 50–59 | 69 (21) | 91 (27) | 46 (13) | 25 (27) | 44 (23) | 38 (19) |
| 60–69 | 73 (23) | 67 (20) | 91 (26) | 18 (19) | 30 (16) | 42 (20) |
| 70–79 | 68 (21) | 92 (27) | 69 (20) | 26 (28) | 67 (35) | 53 (26) |
| ≥80 | 29 (9) | 32 (9) | 18 (5) | 5 (5) | 17 (9) | 18 (9) |
| Years at school | | | | | | |
| <10 | 221 (68) | 244 (71) | 214 (61) | 65 (70) | 151 (79) | 139 (68) |
| ≥10 | 103 (32) | 99 (29) | 135 (39) | 28 (30) | 40 (21) | 66 (32) |
| Post-school education | | | | | | |
| None | 118 (36) | 131 (38) | 100 (29) | 24 (26) | 61 (32) | 48 (23) |
| Short (≤3 years) | 180 (56) | 180 (52) | 195 (56) | 53 (57) | 102 (53) | 112 (55) |
| Long (>3 years) | 26 (8) | 32 (9) | 54 (15) | 16 (17) | 28 (15) | 45 (22) |

[a]Female cases comprised 262 women with invasive and 62 with in situ anal cancer; male cases comprised 87 men with invasive and six with in situ anal cancer. [b]Participation rates were calculated as the proportion of invited subjects who were interviewed. [c]A pseudo-year of diagnosis was assigned to population controls according to the distribution of year of diagnosis among patients of the same sex with anal cancer.

behaviour and venereal diseases and their association with the risk for anal cancer (Frisch et al, 1997). All interviews were conducted by medically trained interviewers who were unaware of the specific study hypotheses. The study was approved by the scientific ethics committees of both participating countries.

We interviewed a total of 417 patients with anal cancer (93 men and 324 women), 534 cancer controls (191 men, 343 women) and 554 population controls (205 men, 349 women). Participation rates and selected characteristics for the study participants are presented in Table 1.

### Tissue analyses

Paraffin-embedded anal cancer specimens were collected from over 60 pathology laboratories in Denmark and Sweden. Specimens were subjected to general primer GP5+/6+ mediated polymerase chain reaction (PCR) analyses for the presence of most, if not all, mucosotropic human papillomaviruses as described elsewhere (Frisch et al, 1997; Jacobs et al, 1997). For the purpose of the present analysis, tumours were divided into those that were positive to one or more of 14 distinct hrHPV types, i.e. types 16, 18, 31, 33, 35, 39, 45, 51, 52, 56, 58, 59, 66 and 68, and those that were negative to each of these hrHPV types.

### Statistical methods

Men and women were analysed separately. Univariate odds ratios (ORs) were calculated with adjustment only for age (<40, 40–49, 50–59, 60–69, 70–79, ≥80 years), country (Denmark, Sweden) and year of diagnosis (1991, 1992, 1993, 1994–95). A pseudo-year of diagnosis was attributed to population controls according to the distribution of year of diagnosis among cases of the same sex. Multivariate logistic regression analyses were performed to identify independent predictors of the risk. Because univariate ORs obtained with the two control groups were generally similar, and because the distribution of major sexual and venereal confounder variables was previously found to be similar in the two control groups (Frisch et al, 1997), we combined them to increase statistical power in the multivariate analyses. The multivariate OR for each variable was adjusted for potential confounding by age at diagnosis (<40, 40–49, 50–59, 60–69, 70–79, ≥80 years), country (Denmark, Sweden), year of diagnosis (1991, 1992, 1993, 1994–95), years at school (<10, ≥10), years of post-school education (none, ≤3, >3), smoking status (current, former, never) and for major sexual and venereal factors. In men, sexual and venereal factors included marital status (ever vs never married) and lifetime number of female partners (0, 1, 2 or 3, 4–9, ≥10) as well as for anogenital warts (yes/no), gonorrhoea (yes/no) and syphilis or hepatitis (yes/no). In women, sexual and venereal factors included marital status (ever vs never married), lifetime number of male partners (0, 1, 2 or 3, 4–9, ≥10), practice of anal intercourse (yes/no), anogenital warts (yes/no), gonorrhoea (yes/no) and history of a sexually transmitted disease in the male partner (yes/no). All regression analyses were performed using likelihood ratio tests by means of the GENMOD procedure in SAS. In tests for trend, we treated categorized continuous variables as continuous variables with the median in each category as the category value (SAS Institute Inc., 1996).

Finally, we performed a set of analyses within the subgroup of 84 men and 304 women with anal cancer for whom we had

© Cancer Research Campaign 1998 

**Table 2** Benign anal lesions[a] and anal suppository use, odds ratios (anal cancer cases vs all controls) and 95% confidence intervals (CI), Danish–Swedish anal cancer case–control study, 1991–94

| | Women | | | | | Men | | | | |
|---|---|---|---|---|---|---|---|---|---|---|
| | Anal cancer cases No. (%) | Rectal cancer controls No. (%) | Population controls No. (%) | Univariate[b] odds ratio (95% CI) | Multivariate[b] odds ratio (95% CI) | Anal cancer cases No. (%) | Rectal cancer controls No. (%) | Population controls No. (%) | Univariate[b] odds ratio (95% CI) | Multivariate[b] odds ratio (95% CI) |
| Anal fissure/fistula | 39 (13) | 53 (16) | 50 (15) | 0.8 (0.6–1.2) | 0.8 (0.5–1.2) | 21 (23) | 20 (11) | 29 (15) | 2.2 (1.2–3.9) | 1.9 (0.97–3.7) |
| Haemorrhoids | 128 (41) | 153 (47) | 148 (44) | 0.8 (0.6–1.1) | 0.8 (0.6–1.1) | 43 (48) | 74 (40) | 74 (37) | 1.6 (0.97–2.5) | 1.8 (1.04–3.2) |
| Anorectal abscess | 11 (3) | 7 (2) | 2 (1) | 2.8 (1.1–6.9) | 3.0 (1.1–8.3) | 8 (9) | 11 (6) | 15 (7) | 1.3 (0.6–3.0) | 1.1 (0.4–2.9) |
| Anorectal prolapse | 12 (4) | 17 (5) | 17 (5) | 0.7 (0.4–1.4) | 0.7 (0.3–1.4) | 6 (7) | 3 (2) | 5 (2) | 3.6 (1.2–11.0) | 2.9 (0.8–10.4) |
| Unspecific anal irritation[c] | 26 (8) | 26 (8) | 19 (5) | 1.3 (0.8–2.2) | 1.1 (0.6–1.9) | 28 (30) | 19 (10) | 22 (11) | 3.8 (2.2–6.8) | 4.5 (2.3–8.7) |
| Anal suppository use | 124 (38) | 103 (30) | 112 (32) | 1.4 (1.1–1.9) | 1.5 (1.1–2.0) | 36 (39) | 66 (35) | 62 (30) | 1.4 (0.8–2.2) | 1.1 (0.6–2.0) |

[a]Only anal lesions that preceded the time of diagnosis (or a similar point in time for population controls) by >5 were considered. [b]Univariate odds ratios are adjusted for age (<40, 40–49, 50–59, 60–69, 70–79, >80 years), year of diagnosis (1991, 1992, 1993, 1994–95) (pseudo-year for population controls) and country (Denmark, Sweden). Multivariate odds ratios are adjusted for age (<40, 40–49, 50–59, 60–69, 70–79, ≥80 years), year of diagnosis (1991, 1992, 1993, 1994–95), country (Denmark, Sweden), years at school (<10, >10), years of post-school education (none, <3, >3), smoker status (current, former, never), and a number of sexual and venereal variables. In men, sexual and venereal variable included marital status (ever married, never married), lifetime number of female partners (0, 1, 2 or 3, 4–9, ≥10), anogenital warts (yes/no), gonorrhoea (yes/no) and syphilis or hepatitis (yes/no). In women, sexual and venereal variables included marital status (ever married, never married), lifetime number of male partners (0, 1, 2 or 3, 4–9, >10), anal intercourse (yes/no), anogenital warts (yes/no), gonorrhoea (yes/no) and history of a sexually transmitted disease in the spouse (yes/no). Respondents who did not report a history of the anal inflammatory lesion in question at least 5 years before the time of diagnosis (or a similar point in the time for population controls) or the use of anal suppositories served as the reference group. [c]Unspecific anal irritation was present when respondents reported a history of anal eczema, anal pruritus or anal moniliasis >5 years before the time of diagnosis (or a similar point in the time for population controls).

performed successful PCR analyses for hrHPV. Possible differences in the distribution of the anal lesions studied between anal cancers that were hrHPV positive (272 women, 55 men) and negative (32 women, 29 men) were evaluated by means of likelihood ratio $\chi^2$-test or Fisher's exact test (two-tailed).

### RESULTS

We considered only anal lesions that preceded the time of diagnosis by more than 5 years to minimize problems in distinguishing these lesions from early symptoms of anorectal cancer. Except for anal abscess, a rare event reported by 3% of female cases ($n$ = 11) and 1% of female controls ($n$ = 9), a history of benign anal lesion was not more common among women with anal cancer (Table 2). In men, however, fissure or fistula, haemorrhoids, anorectal prolapse and unspecific anal irritation – but not anal abscess – were all associated with increased risk (Table 2). Exclusion of men who reported any homosexual experience ($n$ = 14, all cases) did not substantially change estimates of the relative risk. In this restricted analysis among men, multivariate associations with anal fissure or fistula (OR = 1.7; 95% CI 0.8–3.5) and with anorectal prolapse (OR = 2.3; 95% CI 0.6–9.2) became slightly weaker, but associations with haemorrhoids (OR = 2.0; 95% CI 1.1–3.6) and unspecific anal irritation (OR = 4.7; 95% CI 2.4–9.5) remained significantly above unity. A history of inflammatory bowel disease was not associated with the risk for anal cancer in either sex. Only six women (univariate OR = 0.4) and four men (univariate OR = 1.5) reported a history of ulcerative colitis, and four women had Crohn's disease (univariate OR = 0.7). Women, but not men, with anal cancer were significantly more likely to report the use of anal suppositories than controls (multivariate OR = 1.5; 95% CI 1.1–2.0) (Table 2).

We subsequently examined histories of benign anal lesions among case patients stratified according to the viral status of the anal cancer (Table 3). Among case women who reported a history of anal fissure or fistula, all patients who had tumour tissue examined ($n$ = 35) were hrHPV positive. Among case women without prior anal fissure or fistula ($n$ = 219), only 88% were hrHPV postive (Fisher's test, $P$ = 0.02). A similar tendency was present among men; among male cases reporting a history of anal fissure or fistula ($n$ = 15) 83% were hrHPV positive vs 62% among male cases with no prior history of anal fissure or fistula (Fisher's test, $P$ = 0.10). A comparison of anal cancer patients with and without a history of anorectal prolapse or, among men, anorectal abscess showed that case patients with such prior anal lesions were hrHPV positive more often, although not significantly so, than those without such prior lesions. Detectable hrHPV in the anal cancer was equally present in case patients with and without histories of haemorrhoids, unspecific anal irritation or, among women, anorectal abscess (Table 3).

Male cases who had used anal suppositories had detectable hrHPV in their tumour tissue more often than those who did not report such use ($P$ = 0.03), whereas the majority of female cases, irrespective of anal suppository use, were hrHPV positive (Table 3).

### DISCUSSION

Infection with hrHPV is now considered necessary for the development of most, if not all, squamous cell cancers of the uterine cervix (International Agency for Research on Cancer, 1995; Shah, 1997). There is also mounting evidence that the same types of HPV, particularly type 16, are involved in the aetiology of most anal epidermoid carcinomas (Daling et al, 1992; Holm et al, 1994; Frisch et al, 1997). However, other factors involved in the development of anal cancer are poorly characterized. Such factors could either act independently or modify the oncogenic effect of hrHPV. For decades anal inflammation has been considered to be such a contributing factor (Brofeldt, 1927; Buckwalter and Jurayj, 1957),

*© Cancer Research Campaign 1998*

**Table 3** Benign anal lesions[a] and anal suppository use in patients with high-risk human papillomavirus[b]-positive (hrHPV+) and -negative (hrHPV–) anal cancer, Danish–Swedish anal cancer case–control study, 1991–94

| | Women with anal cancer No. (%) | | | Men with anal cancer No. (%) | | |
|---|---|---|---|---|---|---|
| | hrHPV+ | hrHPV– | (*P*-value[c]) | hrHPV+ | hrHPV– | (*P*-value[c]) |
| Anal fissure/fistula | | | | | | |
| Yes | 35 (100) | 0 (0) | | 15 (83) | 3 (17) | |
| No | 219 (88) | 31 (12) | (0.02) | 40 (62) | 25 (38) | (0.10) |
| Haemorrhoids | | | | | | |
| Yes | 107 (90) | 12 (10) | | 25 (64) | 14 (36) | |
| No | 156 (89) | 20 (11) | (0.73) | 29 (67) | 14 (33) | (0.75) |
| Anorectal abscess | | | | | | |
| Yes | 10 (91) | 1 (9) | | 6 (75) | 2 (25) | |
| No | 256 (89) | 31 (11) | (1.00) | 49 (64) | 27 (36) | (0.71) |
| Anorectal prolapse | | | | | | |
| Yes | 11 (100) | 0 (0) | | 5 (100) | 0 (0) | |
| No | 260 (89) | 31 (11) | (0.61) | 48 (63) | 29 (37) | (0.16) |
| Unspecific anal irritation[d] | | | | | | |
| Yes | 23 (92) | 2 (8) | | 16 (64) | 9 (36) | |
| No | 244 (89) | 29 (11) | (1.00) | 38 (66) | 20 (34) | (0.89) |
| Anal suppository use | | | | | | |
| Yes | 104 (90) | 12 (10) | | 24 (80) | 6 (20) | |
| No | 168 (89) | 20 (11) | (0.94) | 31 (57) | 23 (43) | (0.03) |

[a]Only anal lesions that preceded the time of anal cancer diagnosis by >5 years were considered. [b]High-risk HPV positive anal cancers were positive to one or more of HPV types 16, 18, 31, 33, 35, 39, 45, 51, 52, 56, 58, 59, 66 and 68. [c]Statistical tests used were Fisher's exact test (two-tailed) when there were ≤5 observations in one or more cells; otherwise likelihood ratio $\chi^2$-test was used. [d]Unspecific anal irritation was present when respondents reported a history of anal eczema, anal pruritus or anal moniliasis >5 years before the time of diagnosis.

and results from one case–control study have encouraged this belief (Holly et al, 1989). However, there are inconsistencies between the few previous studies as to what specific anal lesions are risk factors. Holly et al, (EA Holly personal communication) found increased risks associated with haemorrhoids and anal fissure/fistula in a combined analysis of women and heterosexual men, but unlike for women in the present study, there was no association with anorectal abscess in either sex. Another case–control study found no significant association with fissures, fistulae or haemorrhoids and the risk for anal cancer in women (Holmes et al, 1988), while a third study did not present data on benign anal lesions (Daling et al, 1987).

One limitation of our and previous case–control studies is that self-reported anal lesions may be subject to differential misclassification. It is well established that early symptoms of anal cancer may be difficult to distinguish clinically from haemorrhoids and other benign conditions (Jensen et al, 1987). We sought to minimize this potential bias by disregarding the 5 years before diagnosis. As in a previous study (Holly et al, 1989), we observed a number of associations with benign anal lesions, but the lack of consistency between the two sexes detracts from the idea of causality, because it is not plausible that benign anal lesions are carcinogenic only in one gender. Anal abscess was associated with risk only in women, whereas a history of haemorrhoids increased the risk only in men. Moreover, it is not conceivable that unspecific anal irritation due to eczema, pruritus or moniliasis in the anal region should be a genuine carcinogenic factor in men only. Rather, we speculate that anal irritation and other benign anal lesions may be linked to one of the major risk factors for anal cancer in men for which it is possible that accurate information has not been obtained from all participants. We have recently shown strong statistical associations between a number of sensitive variables, including the number of partners of the opposite sex and the practice of heterosexual anal intercourse (Frisch et al, 1997). However, not even one out of 396 male controls reported ever having engaged in homosexual activity. Consequently, homosexual experience may be underestimated in the present study because of underreporting and/or self-selection among participants. Underreporting may create spurious associations between anal cancer and other, less sensitive, variables that are associated with homosexual activity. As traumatic and inflammatory anal complaints are reported to be common in homosexual men (Kazal et al, 1976), we suggest that confounding by unreported homosexual experience may – at least in part – account for the significant associations observed between benign anal lesions and anal cancer in men.

The idea that benign anal lesions or the inflammatory process that may accompany such lesions is involved in the causation of anal cancer was also challenged in two recent cohort studies. A follow-up study of 68 000 patients hospitalized in Denmark for haemorrhoids, fissures, fistulae or anal abscesses did not support a causal association. The risk for anal cancer was elevated only in the first few years, but not 5 or more years after the benign anal lesion (Frisch et al, 1994). These temporal associations were confirmed in a subsequent study (Lin et al, 1995). The absence of any convincing link between benign anal lesions and the risk for anal cancer in women (who constituted 78% of the case patients in this study), and the likelihood of a non-causal explanation for the statistical associations observed in men, in combination with the results from previous studies (Holmes et al, 1988; Frisch et al,

© Cancer Research Campaign 1998

1994; Lin et al, 1995), argue against anal inflammation as a genuine aetiological factor.

Nevertheless, in both men and women, we observed a higher proportion of hrHPV-positive anal cancers among patients who reported a history of anal fissure or fistula. Although we cannot rule out the possibility that hrHPV-infected anal mucosa might be more prone to the development of crack sores, we suggest the reverse causal pathway, namely that fissures or fistulae may provide direct viral access to basal mucosal layers. A similar mechanism has been proposed for hrHPV-related cervical neoplasia (Schneider and Koutsky, 1992).

In the aetiology of vulval cancer, hrHPV appears to be involved mainly in cancers histologically categorized as either warty or basaloid carcinoma; for keratinizing squamous cell carcinomas that are not linked to hrHPV to the same extent, unspecific dystrophic or inflammatory vulval lesions may be involved (Toki et al, 1991; Trimble et al, 1996). By analogy, we examined whether anal cancers in which hrHPV was not detected were linked to benign anal lesions and unspecific irritation more firmly than anal cancers testing positive to hrHPV. This was not the case in as much as anal cancer patients reporting such prior anal complaints contained hrHPV DNA in their tumour to a similar or even greater extent than anal cancer patients without prior anal lesions. Moreover, in accordance with other investigations (Holmes et al, 1988; Frisch et al, 1994), the present study failed to support causal speculations based on anecdotal reports of anal cancer in patients with large bowel inflammatory diseases, notably Crohn's disease (Slater et al, 1984).

In conclusion, we suggest that anal fissure or fistula may facilitate viral access to basal epithelial layers. However, benign anal lesions are unlikely candidates both as promoters in hrHPV-related anal carcinogenesis and as causal factors in hrHPV-unassociated anal cancer. Ulcerative colitis and Crohn's disease were not supported as causal factors for anal cancer.

### ACKNOWLEDGEMENTS

This study was supported by grants from the Danish Cancer Society (Nos. 90-7620 and 94-004) and from the Swedish Cancer Society (No. 3258-B95-04XCC). The assistance from pathology, surgery, oncology and gynaecology departments and from private practitioners throughout Denmark and Sweden is gratefully appreciated.

### REFERENCES

Brofeldt SA (1927) Zur Pathogenese des Plattenepithelkrebses der Pars analis recti. *Acta Soc Med Fenn Duodecim* **8**: 3–15

Buckwalter JA and Jurayj MN (1957) Relationship of chronic anorectal disease to carcinoma. *Arch Surg* **75**: 352–361

Daling JR, Weiss NS, Hislop TG, Maden C, Coates RJ, Sherman KJ, Ashley RL, Beagrie M, Ryan JA and Corey L (1987) Sexual practices, sexually transmitted diseases, and the incidence of anal cancer. *N Engl J Med* **317**: 973–977

Daling JR, Sherman KJ, Hislop TG, Maden C, Mandelson MT, Beckmann AM, Weiss NS (1992) Cigarette smoking and the risk of ano-genital cancer. *Am J Epidemiol* **135**: 180–189

Frisch M, Melbye M and Møller H (1993) Trends in incidence of anal cancer in Denmark. *Br Med J* **306**: 419–422

Frisch M, Olsen JH, Bautz A and Melbye M (1994) Benign anal lesions and the risk of anal cancer. *N Engl J Med* **331**: 300–302

Frisch M, Glimelius B, van den Brule AJC, Wohlfahrt J, Meijer CJLM, Walboomers JMM, Goldman S, Svensson C, Adami H-O and Melbye M (1997) Sexually transmitted infection as a cause of anal cancer. *N Engl J Med* **337**: 1350–1358

Goldman S, Glimelius B, Nilsson B and Påhlman L (1989) Incidence of anal epidermoid carcinoma in Sweden 1970–1984. *Acta Chir Scand* **155**: 191–197

Holly EA, Whittemore AS, Aston DA, Ahn DK, Nickoloff BJ and Kristiansen JJ (1989) Anal cancer incidence: genital warts, anal fissure or fistula, hemorrhoids, and smoking. *J Natl Cancer Inst* **81**: 726–731

Holm R, Tanum G, Karlsen F and Nesland JM (1994) Prevalence and physical state of human papillomavirus DNA in anal carcinomas. *Mod Pathol* **7**: 449–453

Holmes F, Borek D, Owen-Kummer M, Hassanein R, Fishback J, Behbehani A, Baker A and Holmes G (1988) Anal cancer in women. *Gastroenterology* **95**: 107–111

International Agency for Research on Cancer (1995) Human Papillomaviruses. IARC Monographs on the Evaluation of Carcinogenic Risks to Humans, Vol. 64. World Health Organization: Lyon

Jacobs MV, Snijders PJF, van den Brule AJC, Helmerhorst THJM, Meijer CJLM and Walboomers JMM (1997) A general primer GP5+/GP6+ mediated PCR-enzyme immunoassay method for rapid detection of 14 high risk and 6 low risk human papillomavirus genotypes in cervical scrapings. *J Clin Microbiol* **35**: 791–795

Jensen SL, Hagen K, Shokouh-Amiri MH and Nielsen OV (1987) Does an erroneous diagnosis of squamous cell carcinoma of the anal canal and anal margin at first physician visit influence prognosis? *Dis Colon Rectum* **30**: 345–351

Kazal HL, Sohn N, Carrasco JI, Robilotti JG Jr and Delaney WE (1976) The gay bowel syndrome: clinico-pathologic correlation in 260 cases. *Ann Clin Lab Sci* **6**: 184–192

Lin AY, Gridley G and Tucker M (1995) Benign anal lesions and anal cancer. *N Engl J Med* **332**: 190–191

Melbye M, Rabkin CS, Frisch M and Biggar RJ (1994) Changing patterns of anal cancer incidence in the United States, 1940–89. *Am J Epidemiol* **139**: 772–780

SAS Institute Inc. (1996) The GENMOD Procedure. In: SAS/STAT© Software: Changes and Enhancements through Release 6.11, pp. 231–316. SAS Institute Inc.: Cary, NC

Schneider A and Koutsky LA (1992) Natural history and epidemiological features of genital HPV infection. In *The Epidemiology of Human Papillomavirus and Cervical Cancer*, IARC Scientific Publications No. 119, Muñoz N, Bosch FX, Shah KV, Meheus A (eds), pp. 25–52. International Agency for Research on Cancer: Lyon

Shah KV (1997) Human papillomaviruses and anogenital cancers. *N Engl J Med* **337**: 1386–1388

Slater G, Greenstein A and Aufses AH (1984) Anal carcinoma in patients with Crohn's disease. *Ann Surg* **199**: 348–350

Toki T, Kurman RJ, Park JS, Kessis T, Daniel RW and Shah KV (1991) Probable non-papillomavirus etiology of squamous cell carcinoma of the vulva in older women: a clinicopathologic study using in situ hybridization and polymerase chain reaction. *Int J Gynecol Pathol* **10**: 107–125

Trimble CL, Hildesheim A, Brinton LA, Shah KV and Kurman RJ (1996) Heterogenous etiology of squamous carcinoma of the vulva. *Obstet Gynecol*, **87**: 59–64

© Cancer Research Campaign 1998